# DNA Damage/Repair Management in Cancers

**DOI:** 10.3390/cancers12041050

**Published:** 2020-04-23

**Authors:** Jehad F. Alhmoud, John F. Woolley, Ala-Eddin Al Moustafa, Mohammed Imad Malki

**Affiliations:** 1 Department of Medical Laboratory Sciences, Faculty of Applied Medical Sciences, Al-Ahliyya Amman University, Amman 19328, Jordan; 2 Department of Molecular & Clinical Pharmacology, Liverpool University, Liverpool L69 3GE, UK; john.woolley@liverpool.ac.uk; 3 College of Medicine, QU Health, Qatar University, Doha P. O. Box 2713, Qatar; aalmoustafa@qu.edu.qa

**Keywords:** DNA damage, DNA repair pathway, DNA lesion, genomic instability

## Abstract

DNA damage is well recognized as a critical factor in cancer development and progression. DNA lesions create an abnormal nucleotide or nucleotide fragment, causing a break in one or both chains of the DNA strand. When DNA damage occurs, the possibility of generated mutations increases. Genomic instability is one of the most important factors that lead to cancer development. DNA repair pathways perform the essential role of correcting the DNA lesions that occur from DNA damaging agents or carcinogens, thus maintaining genomic stability. Inefficient DNA repair is a critical driving force behind cancer establishment, progression and evolution. A thorough understanding of DNA repair mechanisms in cancer will allow for better therapeutic intervention. In this review we will discuss the relationship between DNA damage/repair mechanisms and cancer, and how we can target these pathways.

## 1. Introduction

DNA damage can alter nucleotide sequences and lead to expression of dysfunctional proteins that impact normal cellular physiology. Sources of DNA damage can be endogenous or exogenous and include reactive oxygen species (ROS) or ionizing radiation [1]. DNA damaging agents can broadly be classified into two different categories: clastogens and aneugens. Clastogens cause chromosomal breaks and induce micronuclei (MN) due to generation of acentric chromosomal fragments. In contrast, aneugens lead to the incorporation of whole chromosomes in MN by generation of aneuploidy that affects cell proliferation and the mitotic spindle apparatus [2].

Genotoxic agents cause structural changes in DNA by disrupting covalent bonds between nucleotides, preventing accurate replication of the genome [3]. Significant numbers of cells in the human body are subjected to DNA damage on a continuous basis which leads to alterations in genome replication and transcription. Although the DNA repair machinery can correct some of these lesions, unrepaired or misrepaired DNA can lead to genome aberrations and mutations that affect cellular function [4]. Genetic defects, especially those occurring in oncogenes, tumor-suppressor genes, genes that control the cell cycle, etc., can impact cell survival or proliferation [5]. Such DNA damage can be carcinogenic [6]. DNA repair proteins trigger checkpoints to recognize sites of damage and either activate corrective pathways or induce apoptosis [7].

Endogenous agents induce replication stress or generate free radicals derived from the oxidative metabolism, whereas exogenous agents such as ionizing or ultraviolet (UV) radiation and chemotherapy induce structural changes such as single strand (SSB) or double strand breaks (DSB) in DNA via base modifications, helix-distorting bulky lesions, or cross-links of DNA strands, and are repaired by biochemically distinct DNA repair pathways [8]. DSBs are the most severe form of DNA damage in eukaryotic cells, because they lead to inefficient repair and cause mutations or induce cell death.

## 2. Types of DNA Damage

DNA lesions affect a huge number of cells in the human body, occuring at a rate of 10,000 to 1,000,000 molecular lesions per cell per day [9]. Unrepaired or incorrectly repaired DNA damage can lead to serious genome aberrations or mutations, potentially affecting cell survival [4]. However, some mutations change cell proliferation due to defects of certain genes, e.g., oncogene, a tumor-suppressor gene, or a gene that controls the cell cycle.

**Figure 1:**
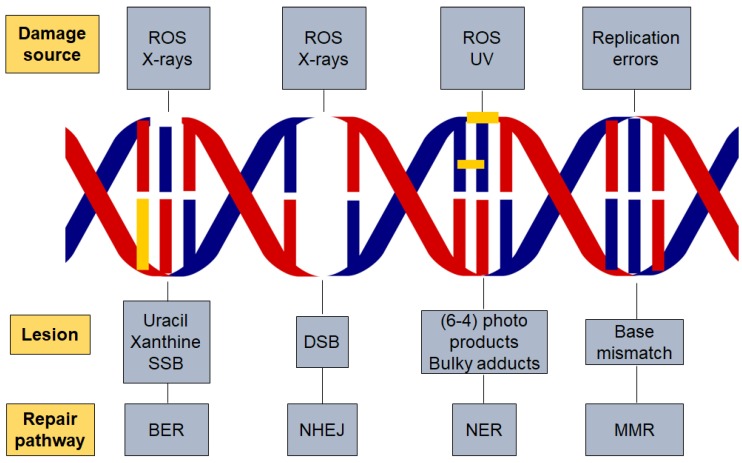
DNA damage and repair pathways. Different factors are responsible for initiating DNA damage such as radiation and reactive oxygen species which cause several types of lesions in the DNA double helix. The repair pathway involved in the process is dependent on the damaging agent and lesion generated. Base excision repair (BER), nucleotide excision repair (NER), non-homologous end joining (NHEJ), reactive oxygen species (ROS) and DNA mismatch repair (MMR).

One of the main sources of DNA damage is ionizing irradiation, which can cause direct or indirect DNA damage leading to changes in the structure of DNA that affects nuclear stability [10]. Ionizing radiation can be of various types such as alpha particles, beta particles or gamma radiation [11]. This radiation releases energy when passing through cellular material and can disrupt proteins and nucleic acids [12]. Irradiation can cause DSB at the phosphodiester backbone of DNA [13]. The level and complexity of DNA damage is influenced by the dose of radiation. Radiation doses can also impact the cellular microenvironment and the type of DNA damage [14]. In addition, other factors play a role in initiating DNA damage, such as reactive oxygen species. Radiation damages cells by direct ionization of DNA and other cellular targets and by indirect effects through ROS [15]. Oxygen-derived free radicals in the tissue environment are produced due to the exposure to ionizing radiation; these include hydroxyl radicals, superoxide anion radicals and hydrogen peroxide. Two-thirds of the damage caused by X-rays and gamma rays are efficient in killing cancer cells. Radiotherapy leads to the production of ROS which affect the survival rate and increase the level of apoptosis in normal cells (Figure 1) [16].

After DNA damage occurs, the DNA repair proteins should identify the site of the damage and determine whether to repair the damage or push the cells towards apoptosis through a DNA damage check point. Apoptosis or programmed cell death (PCD) plays a vital role in maintaining tissue homeostasis by removing diseased or injured cells. Mitochondrial fragmentation within such cells leads to caspase activation and cell death when cells pass through critical checkpoints [17,18]. Conversely, survival pathways such as target of rapamycin complex 1 (TORC1) are activated in response to genotoxic stress to maintain deoxynucleoside triphosphate pools.

Abnormal cell proliferation is one of the hallmarks of cancer [19], but the behavior and the response of cancer cells to the treatments is not well known and still under investigation [20].

## 3. DNA Damage Response

DNA repair pathways are encoded by a class of proteins that detect DNA double stand breaks, chromosomal fragmentation, translocation and deletions, and can correct some alterations [21]. Cells suffer constant and regular insults from genotoxic agents. The DNA damage response (DDR) pathway responds to cellular damage by using signal sensors, transducers and effectors [22]. Such mechanisms help the genome to tolerate or correct damage on an ongoing basis. Endogenous cellular processes produce free radicals, which affect human cells around 10,000 times/day and cause oxidative DNA damage [23]. The presence of DNA damage or DNA replication stress leads to abnormalities in DNA structure which subsequently stimulate the DDR pathway [24].

DDR mechanisms include multiple DNA repair pathways, damage tolerance processes and cell-cycle checkpoints [25]. DNA replication stress activates DDR leading to DNA double-strand breaks (DSBs) and genomic instability [26]. DDR can regulate genomic stability by repairing damaged DNA or removing defective cells by programmed cell death [27]. On the other hand, genomic instability and deregulation of DNA damage repair (DDR) pathways can be associated with cancer progression [28]. Mutations or deletion of genes responsible for regulating cell division or tumor suppressors can also lead to genomic instability and cancer [29]. Genetic alterations that lead to cancer are more likely to occur in actively proliferating tissues. Cells with high rates of proliferation are more susceptible to DNA damage and tumorigenesis [30]. Genomic instability is responsible for tumor progression and the modification of normal cells to cancer cells. In hereditary cancers, the frequency of the mutated base pair is induced due to loss in the function of the DNA repair genes [31,32].

The tumor-suppressor protein Tp53 identifies the presence of DSB and activates the signaling pathways that regulate tumor progression and promote apoptosis. Mutations in the p53 gene affect DNA damage repair and promote cancers [33]. A functional DDR is essential for human health, and dysfunction can lead to several diseases such as immune deficiency, neurodegeneration, premature aging, and cancer [25]. The PIKK kinase family members, ataxia telangiectasia mutated (ATM) and RAD3-related (ATR) are major regulators of DDR. They are sensor proteins and often work together in response to DNA damage signals [24]. ATM and ATR recognize changes in the DNA structure and, as a consequence, mediate downstream protein phosphorylation events and facilitate DDR [34].

Well-studied DNA repair pathways including base excision repair (BER) for SSBs, nucleotide excision repair (NER) for bulky adducts, and non-homologous end joining (NHEJ) and homologous recombination (HR) for DSBs. In addition, there is also DNA mismatch repair (MMR) for the correction of replication errors such as base-pair mismatches and loops/bubbles arising from a series of mismatches [35].

## 4. Components of the DNA Damage Response

In mammalian cells, the major DDR-signaling components include the protein kinases ATM and ATR, which are induced by DSBs and replication protein A (RPA) that binds to single-stranded DNA (ssDNA) [36,37]. The protein kinases CHK1 and CHK2T are targeted by ATM/ATR. Both are responsible for inhibiting cyclin-dependent kinase (CDK) activity through different mechanisms, which are facilitated by stimulation of the p53 transcription factor [38]. Cell-cycle progression at the G1-S, intra-S and G2-M “cell-cycle checkpoints” are reduced or arrested by the inhibition of CDKs. This step is thought to be essential for improving the chance of DNA repair before replication or mitosis is completed. However, DNA repair is enhanced by ATM/ATR signaling through stimulating DNA repair proteins transcriptionally or post-transcriptionally by modulating their phosphorylation, acetylation or ubiquitylation [39]. The activation of DNA repair proteins acts by recruiting repair factors to the site of damaged DNA. Proteomics studies demonstrate that the DDR regulates additional cellular processes, as this technique recognizes a considerable amount of uncharacterized ATM/ATR-mediated phosphorylation sites [40]. In the event that there is no defect in this mechanism, this will allow efficient DNA repair so that DDR inactivation ensues, which leads to retrieving normal cell functions. On the other hand, if the repairing mechanism is not able to eliminate the damaged DNA, chronic DDR signaling prompts cell apoptosis, causing cell death or a state of stable cell-cycle arrest; both responses function as a potential anti-tumor response [41].

Furthermore, DDR is affected by chromatin structures that may be modified in response to DNA damage [42]. One of the main examples is the phosphorylation of serine-139 of the histone H2A variant. H2AX is mediated by ATM/ATR/DNA-PK, on chromatin located on the sites of the DSB. The stimulation of DSB repair and increased DSB signaling are through the ubiquitin-adduct formation in the DNA damaged regions, and the recruitment of DDR factors besides other chromatin-modifying components [39]. Remarkably, the chromatin relaxation at sites of DSBs is caused due to the activation of ATM [43], and in DDR the H2AX tyrosine-142 phosphorylation is functioning [44,45]. These findings suggest that further investigations are needed on DDR-induced chromatin modifications.

## 5. DDR and Disease Treatment

The most well-known cancer treatments function based on generating DNA damage, such as radiotherapy and chemotherapy. These types of treatments are efficient, although they cause dose-limited toxicities in normal cells. The rapid proliferation of cancer cells compared with normal cells is due to an impaired DDR. However, the rationale underlying the resistance to cancer therapy is associated with common DNA repair mechanisms. For instance, it has been reported that the treatment of glioblastoma is difficult as a result of the unique properties of their DDR machinery [46]. These findings meditated that using DDR inhibition might promote the efficacy of radiotherapy and DNA-damaging chemotherapies. Moreover, there are many DDR-inhibitor drugs in early stage trials including Mitomycin C, Cisplatin, Etoposide (Topo II), anthracyclines, Epirubicin and Daunorubicin (Topo II) [47,48]. Blocking apoptosis is another potential application for DDR inhibitors to reduce the toxicity levels in normal cells, which are mediated by CHK2 and p53 [4].

Generally, one or other aspects of the DDR are defected in all cancer cells due to alterations of the behavior of cells during tumor evolution. Therapeutic outcomes are improved when there is a decrease or loss in the DDR factors. There is an exception of poor resistance to the therapeutic effect in the case of disorders in p53 and other pro-apoptotic proteins [49,50]. However, the use of DDR pathway inhibitors has a greater effect on cancer cells than normal cells. In some cases, different DNA repair pathways could be involved and might overlap in function and each pathway might be used as an alternative pathway in repairing DNA damage. An example of the repairing pathway inhibitors includes drugs that target the enzymes that facilitate the repairing process, such as PARP-1, which binds SSBs and BER intermediates. Remarkably, PARP inhibitors are comparatively non-toxic to normal cells, but impact cytotoxicity in homologous recombination deficiency cells, especially in cells which have a defect in BRCA1 or BRCA2 genes [51,52]. The HR-defective cells are defected in BRCA gene and are considered as cancer cells, indicating that the wild-type BRCA allele is completely absent. In patients having one wild-type BRCA allele and one mutant BRCA allele, the HR is unimpaired in their normal cells. HR is required to repair the accumulation of SSBs that are converted later into pernicious DSBs due to inhibition of PARP1. The BRCA1- or BRCA2-deficient cancer cells are not able to repair the lesions in the same way as the normal cells that are repaired by HR [53]. The HR repair is impaired in cancerous cells, and subsequently the tumor cells lead to apoptosis. These observations prove that the defects occur in two different genes or pathways, together resulting in cell apoptosis, whereas defects in one of the two different genes or pathways do not affect the cell survival [54].

In addition, in a phase I trial on PARP inhibitor as a single agent in patients with BRCA mutations using oral PARP inhibitor olaparib in order to prove the safety of olaparib as a single agent, the patients with BRCA-mutated breast, ovarian, or prostate tumors showed a positive response toward this inhibitor [55]. In the later phase II studies, performed on patients with breast or ovarian cancer with germline BRCA mutations, one-third of them had a positive response to the drug with a low level of toxicities [56]. Currently, PARP inhibitors are used to treat BRCA-mutated ovarian cancer and also have been approved for the treatment of advanced BRCA-mutated breast cancer [57]. Further trials using PARP inhibitors on tumors that have HR defects due to mutation or epigenetic inactivation of HR components suggest the applicability of this treatment to be used for ovarian, prostate and pancreatic cancers. Furthermore, the effectiveness of DNA-damaging agents improved after using CHK1 inhibitors, particularly in p53-deficient cells [58].

The discovery of CRISPR/Cas9 technology, which is based on genome editing, could be performed efficiently through targeting the genes that cause cancers and cancer therapy. A CRISPR/Cas9 system was utilized to target the oncogene HER2 leading to inhibition of cell growth in breast cancer cell lines. The addition of PARP inhibitors increased the inhibitory effect [59]. Poly (ADP-ribose) polymerase (PARP) inhibitors are currently used as cancer treatment only in cells defective in the homologous recombination (HR) pathway [60]. The clinical use of PARP inhibitors might extend especially after recognizing the genetic targets that stimulate or mimic HR deficiencies using CRISPR/Cas9. A study demonstrated that TP53 induced glycolysis and apoptosis regulator (TIGAR) is developed in various types of cancers, and the overall survival in ovarian cancer was decreased when the expression of TIGAR was increased [61]. Therefore, in order to improve the sensitivity of cancer cells to olaparib, TIGAR was knocked down which has an impact on metabolic pathways and increased the cytotoxic effects of olaparib. This step causes downregulation of BRCA1 and the Fanconi anemia pathway and promotes programmed cell death of these cells [62].

Improving the methods to distinguish between cancer and normal cells is necessary for the development of diagnostic procedures which help to ameliorate the efficacy of DNA-damaging and DDR-inhibitor therapies. Moreover, screening for DDR-markers as DDR is activated during oncogenesis, is sensitive and beneficial especially for the detection of cancer that might allow efficient detection of pre-malignant disease [63]. Improving therapeutics that stimulate DDR events can be possible in the longer term to control cancer incidence. This experiment was applied to genetically engineered mice expressing p53-dependent DNA damage responses and showed less tumors compared to wild-type mice [64].

## 6. DNA Repair Pathways

The human DNA is exposed to a huge number of DNA damaging agents every day. Any defect in the process of repairing these lesions might impact translation and transcription, leading to mutations or enormous genome aberrations affecting cell survival or organism life [65]. Failure of DNA repair mechanisms leads to the formation of mutations. Cancer initiates from high genome modifications or DNA aberrations such as deletions, translocations, loss of heterozygosity and amplifications. DNA damage and defects in the repair genes are responsible for the accumulation of mutations and cancers [66]. Cancer development or mutagenesis is highly related to impairment of DNA damage repair. Measuring the levels of DNA damage gives an overview of the level of carcinogenic chemicals leading to tumor genesis during the activation of repairing mechanisms after the damage occurs. Different types of DNA damage are responsible for promoting several DNA aberrations. These mostly occur during replication by causing DNA strand breaks due to ineffective topoisomerase I and topoisomerase II [67]. Furthermore, hydrolytic reactions and non-enzymatic methylations are also responsible for damaging thousands of DNA bases per cell every day [4]. Some factors such as the imbalance between DNA damage and repair also play a critical role in the accumulation of mutations in cancer cells. The frequency of mutation increases proportionally to the increase in the amount of DNA damage and reduction in the DNA repair [68]. In addition, the genome repairing mechanism causes significant changes in the chromatin components when an unprompted reaction influences chromatin and DNA methylation [39,69].

The DNA damage response pathway is activated by cells in response to DNA damage. There are several types of cell responses such as cell-cycle arrest and stimulation of transcriptional and post-transcriptional mechanisms, which induce the genes associated with DNA repair and can activate programmed cell death in certain cases [70]. Replication of genetic information and rearrangement are facilitated by the double-helical structure of DNA. Mostly, the effect of the DNA damage is harmful despite the fact that DNA mutation or recombination is the source of genetic variability and is essential for life after DNA damage. Several mechanisms can be activated to repair damaged DNA including direct repair, base excision repair (BER), nucleotide excision repair (NER), mismatch repair, DNA strand break repair, non-homologous end joining (NHEJ) and homologous recombination (HRR).

### 6.1. Direct Repair

The direct repair mechanism depends on a single protein in eliminating the DNA damage and lesions. However, direct repair is less prone to errors, has efficiency in the maintenance of genetic information, and does not take part in incisions of the sugar-phosphate backbone or base excision [71].

In mammalian cells, the DNA damage that occurs from alkylating agents leads to stimulating the direct repair mechanism to repair the damage. In these cells, two main proteins activate the direct repair mechanism, called “O6-methylguanine-DNA methyltransferase (MGMT)” and “ALKBH family dioxygenases” [72].

Ionizing irradiations, including ultraviolet (UV) light, damage the DNA molecules by generating thymine dimers in the DNA chain between neighboring thymines, causing distortion of the double helix due to the weak hydrogen bond among dimers [73]. Eventually, this defect leads to mutations due to replication errors. DNA photolyase is a light-dependent DNA repair enzyme which protects the cells against this type of DNA damage by binding and removing the thymine dimer from the DNA strand [74].

**Figure 2:**
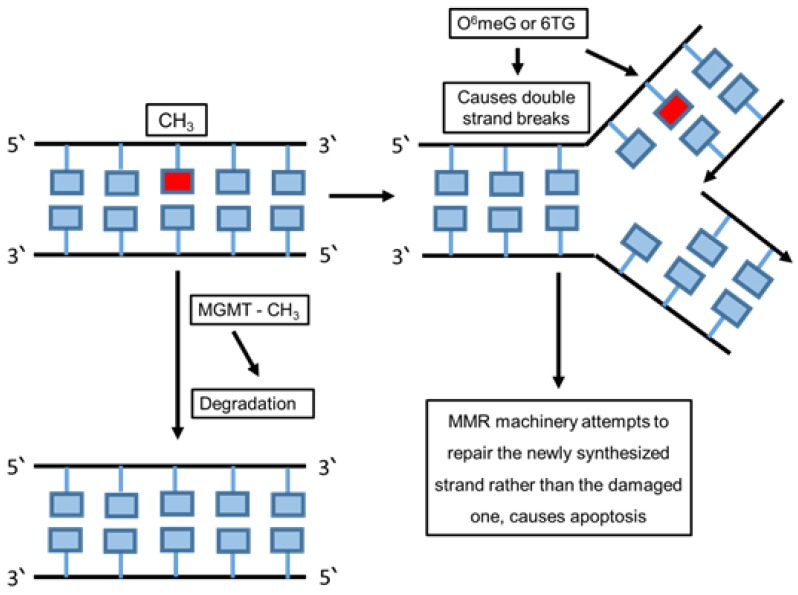
Direct DNA repair pathway. The schematic figure summarizes the direct repair mechanism after the damage on the template DNA strand. This type of repair leads to exclusion of DNA and RNA damage using chemical reversion that does not need a nucleotide template and breakage of the phosphodiester backbone or DNA synthesis.

Most DNA damage is repaired by removing the damaged bases by re-synthesizing and rebuilding the damaged region. The direct reversal mechanism is more effective in repairing specific types of damaged DNA that happen repeatedly, such as exposure to UV light. This leads to pyrimidine dimers and adds methyl or ethyl groups at the O(6) position of the purine ring which cause alkylated guanine residue alteration [75]. This mechanism also replaces the damaged DNA bases chemically by alkalyting agent compounds to transfer methyl groups from the base to a cysteine side chain within the alkyltransferase [76]. However, methylation of the O6 position of guanine causes DNA damage and forms complementary base pairs with thymine instead of cytosine from O6-methylguanine product. The O6-methylguanine methyltransferase (MGMT) enzyme plays a role in repairing this damage in its active site by transferring the methyl group from O6-methylguanine to a cysteine residue [20,77]. It repairs the products caused by the addition of guanine on the O6-position such as O (6)-[4-oxo-4-(3-pyridyl) butyl] guanine and O6-chloroethylguanine. Several factors play critical roles in producing these products such as alkylating (environmental pollutants), carcinogens, methylating (tobacco) and chloroethylating (anticancer drugs) (Figure 2) [78].

### 6.2. Base Excision Repair (BER)

Base excision repair is an essential DNA repair pathway that corrects DNA damage from oxidative, alkylating and deamination events [79,80]. BER induces the DNA damage repair through two common pathways including short patch (repair tract of a single nucleotide) and long patch (repair tract of at least two nucleotides) [81]. The single nucleotide area is repaired by short patch BER pathway, whereas two nucleotide areas or more are repaired by long patch BER pathway. Four proteins are essential for the function of the BER pathway, including DNA glycosylase, AP endonuclease, DNA polymerase and DNA ligase [82].

The main biological function of BER is to remove uracil produced by cytosine deamination from the DNA. In addition, the uracil N-glycosylase (UNG) enzyme eliminates the uracil (U) from both single-stranded DNA (ssDNA) and double-stranded DNA (dsDNA) [83,84]. The BER pathway repairs the damaged DNA throughout the cell cycle [85]. BER is mostly responsible for removing small, non-helix-distorting base lesions from the genome [86]. The BER pathway is activated by DNA repair enzymes such as uracil-DNA glycosylases (UDGs) which are mono-functional glycosylases.

The BER pathway uses mainly DNA glycosylases as the first enzyme to identify the DNA damage and stimulate the elimination of damaged bases [87]. When DNA glycosylases bind with the damaged base, they induce the aberrant base to flip out of the double helix and enter the binding site of the enzyme. This leads to the formation of a protein-substrate complex; then, the glycosylase catalyses the cleavage of the N-glycosidic bond between the substrate base and the 2’-deoxyribose [88]. This step is efficient to remove the damaged base and generate an apurinic/apyrmidinic site (AP site). This site is a particular place in the DNA does not have a purine or pyrimidine base which happens unprompted or due to DNA damage [89].

DNA AP endonuclease or a DNA AP lyase cleaves the DNA backbone, and the activity of AP endonuclease produces a single-stranded DNA nick 5’ to the AP site, while the activity of DNA AP lyase generates parallel nick 3’ to the AP site [88]. In addition, AP endonuclease generates a new nick causing a single-nucleotide gap in the DNA causing 3’-hydroxyl and a 5’-phosphate [90]. The polymerase fills the gap in the DNA by adding the correct nucleotides and the repairing mechanism completes the helical DNA shape, which is maintained after sealing the nick by a DNA ligase [82].

**Figure 3:**
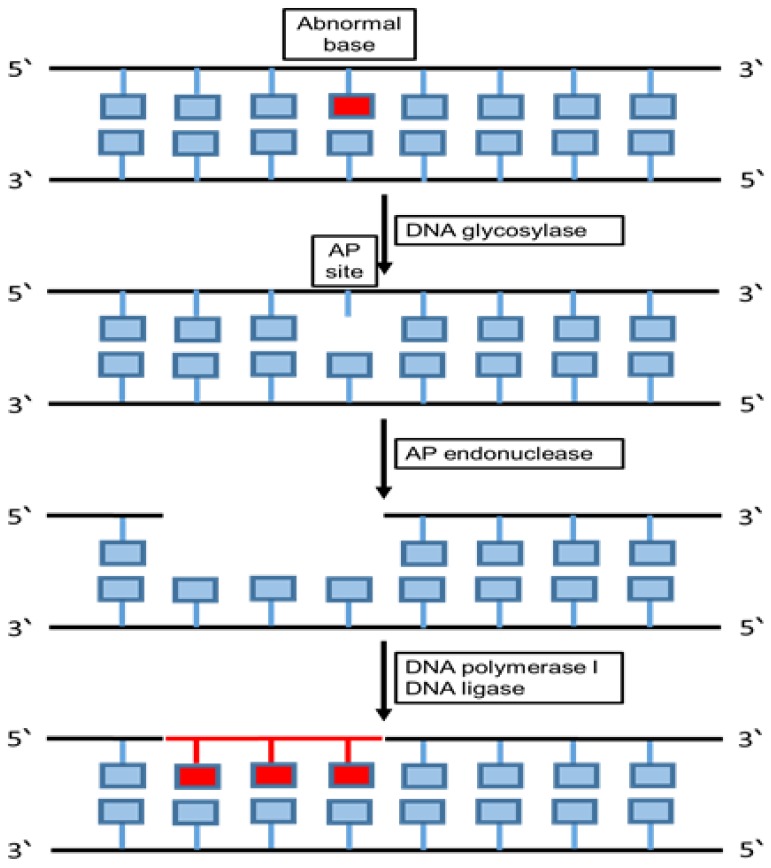
Base excision repair pathway. The schematic diagram summarizes the main components and the mechanism of the base excision repair (BER) pathway. This repairing pathway removes and replaces the faulty DNA segment with a new segment through allowing the cells to eliminate part of a damaged DNA strand and substitute it through DNA synthesis using the undamaged strand as a template.

Pre-mutagenic cytosine (C) damage is eliminated from DNA by human endonuclease III homologue (hNTH1). 5-hydroxycytosine is involved in the BER repair pathway by playing a critical role in improving the possibility of the repairing process using adenine that results in C to T transition mutations [91]. Furthermore, 5-hydroxycytosine (5-OHC) is a cytosine-stable oxidation product associated with high recurrence of C to T transition mutations. Sometimes, the BER pathway fails to identify damage and when this lesion serves as a template during DNA synthesis, replicative DNA polymerases predominantly mis-merge dAMP at the primer terminus, causing mutations which may result in developing later diseases (Figure 3) [92].

### 6.3. Nucleotide Excision Repair (NER)

Nucleotide excision repair (NER) is one of the major DNA repair pathways to protect the cells against DNA lesions that vary structurally and chemically. The most prevalent lesions are produced from additions of bulky covalent adducts initiated by nitrogenous bases and are affected by UV light, ionizing irradiation, electrophilic chemical mutagens, drugs and chemically active endogenous metabolites [93]. 

**Figure 4:**
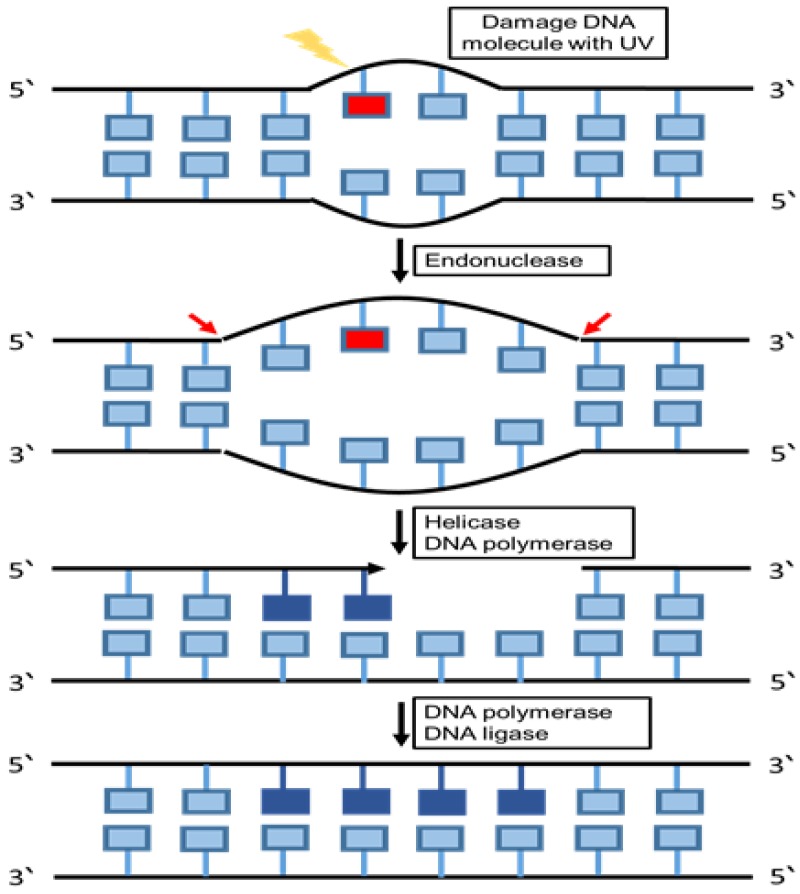
Nucleotide excision repair pathway. The schematic diagram summarizes the main components and the mechanism of the nucleotide excision repair pathway. In this repairing pathway the damaged bases are cut out within a sequence of nucleotides, and replaced with DNA as directed by the undamaged template strand. The nucleotides modified by bulky chemical adducts and pyrimidine dimers formed by UV radiation were removed in this repair system.

NER detects DNA damage through two mechanisms, including global genomic NER (GG NER) and transcription-coupled NER (TC NER). Detection of damaged DNA is an important step in any DNA repairing process. The structural changes in the whole genome are recognized by the GG-NER sub pathway, which repairs the transcribed and un-transcribed DNA strands. The genome is constantly scanned and any disfigurement of the helix will be identified. The TC-NER sub pathway is involved when the damage affects the DNA failure in the NER function, which might lead to UV- sensitivity and a high incidence of cancer [94], for instance, xeroderma pigmentosum (XP), Cockayne syndrome (CS), neurological defects, and trichothiodystrophy (TT D) [95,96]. NER is activated after identifying the damage in order to regulate the level of DNA repair [97]. The NER repairing pathway activated when the double-stranded DNA (dsDNA) occurs in the structure of the DNA, causing a disturbance in the stability of the genetic material. In case of extensive damage in the structure of double-stranded DNA (dsDNA), the BER repairing system replaces with NER substrates in order to repair the DNA aberration [98]. The highly sensitive recognition is essential to detect damage to initiate NER substrates. Compared with BER, the single specialized glycosylas responsible for NER in each process recognize and eliminate the damaged bases at the same time. Furthermore, various proteins are recruited to the damaged complexes with irregular compositions and are involved in a multistep process in NER recognition of the DNA aberrations [99]. NER endonucleases are responsible for eliminating damaged fragments after completing the formation of the pre-incision complex [97]. Several studies discuss the Xeroderma pigmentosum group C proteins (XPC), which play an important role in the initial steps of identifying the damaged DNA and in NER pathway activation [100]. The results of analyzing the damaged substrates reveal that some other factors are considered as a damaging sensor, such as XPA and its complexes with RPA and XPC [101]. A confocal microscopy study showed that XPC might be inactivated after UV damages when the XPA is not existing in the cells, whereas in the absence of XPC in cells, XPA is unable to bind to the damaged site in the DNA [102]. Moreover, biochemical studies found that XPC is essential for the recruitment of other factors necessary for the GG-NER mechanism (Figure 4) [100].

### 6.4. Mismatch Repair (MMR)

DNA mismatch repair is a system for recognizing and repairing erroneous insertions, deletions and mis-incorporations of bases that may arise during DNA replication and recombination, and mismatch repair also repairs some forms of DNA damage. Defects in MMR increase the spontaneous mutation rate, and also induce multistage carcinogenesis [103]. In addition, DNA mismatches generated during the DNA replication can be corrected by the MMR. The MMR pathway prevents permanent mutations in cell divisions. Therefore, any defect in the MMR will increase the unprompted mutational rate. MMR is responsible for minimizing the number of replications associated with errors. Most of the human cancers, whether hereditary or non-hereditary, are linked with the inactivation of MMR in the cells, and some particular DNA damage demands the MMR mechanism to be functioning for cell cycle arrest and/or programmed apoptosis. Therefore, MMR has an important role in the DNA damage response pathway to eradicate the seriously damaged cells and suppress both mutagenesis in the short term and tumorigenesis in the long term [104]. Microsatellites (MS) are mostly identified in various solid and hereditary malignancies. Microsatellite instability (MSI) is a hypermutator phenotype that develops in various tumors via deficiencies in the mismatch repair (MMR) complex, such as hereditary nonpolyposis colorectal cancer syndrome (HNPCC), glioblastomas, lymphomas, and stomach, urinary tract, ovarian and endometrial tumors [105,106]. Chromosome instability generates aneuploidy chromosomes and/or abnormal chromosome structure, which is caused due to increases in the chromosomal mis-segregation in mitosis [107]. As a result, micronuclei will form as an indication of a DNA damage in the cells [108].

**Figure 5:**
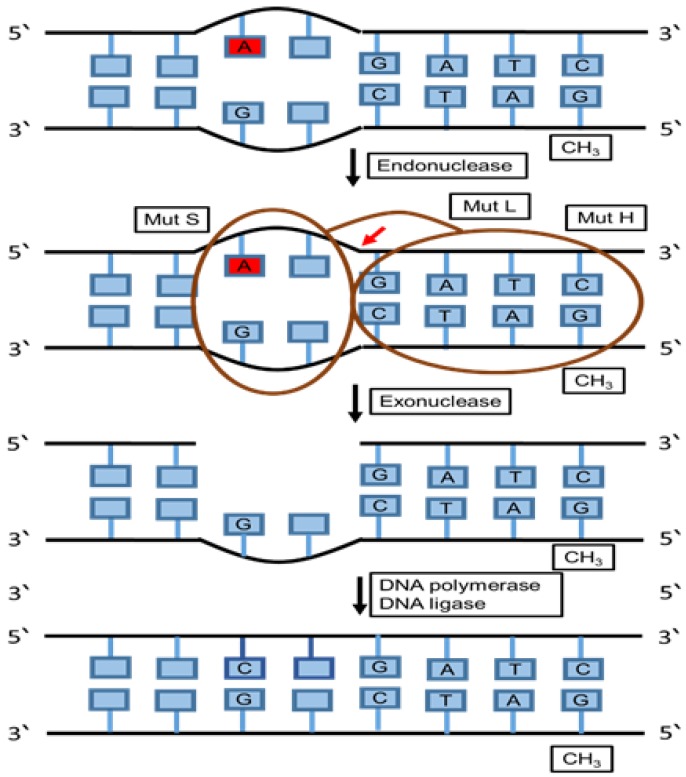
Mismatch repair (MMR) pathway. The schematic diagram summarizes the main components and the mechanism of the mismatch repair pathway. This repairing pathway removes and replaces mispaired bases that were not fixed during proofreading using a group of proteins that recognizes and binds to the mispaired base. Then, other protein complexes chop off the DNA near the mismatch, which is followed by cutting of the incorrect nucleotide and surrounding patches of DNA using specific enzymes in order to be able to replace the missing section with the correct nucleotides.

Human exonuclease 1 (hExo1) is an enzyme involved in MMR. It preserves genomic integrity by the nucleolytic processing of DNA intermediates. A 5’ structure-specific nuclease family of exonucleases and endonucleases also has a hExo1 enzyme which functions in various DNA repair pathways. MMR is responsible for the activation of primary exonuclease. Moreover, during double strand break repair (DSBR), MMR contributes to removing the damaged DNA. Furthermore, hExo1 is essential for enhancing telomere recombination at transcription-induced telomeric structures. On the other hand, hExo1 provides the damaged region with a nick 5′ to produce 5′-3′ hydrolysis on the double-stranded DNA. The activity of 5′–3′ hydrolysis requires the binding of hExo1 with MutS (MMR protein) which is ATP-dependent in a mismatch. Exonucleolytic activity is regulated by the interaction between hExo1 and several MMR proteins such as MutL and the DNA lesion recognition proteins MutSα and MutSβ (Figure 5) [109,110].

### 6.5. Non-Homologous End Joining (NHEJ) and Homologous Recombination (HRR)

**Figure 6:**
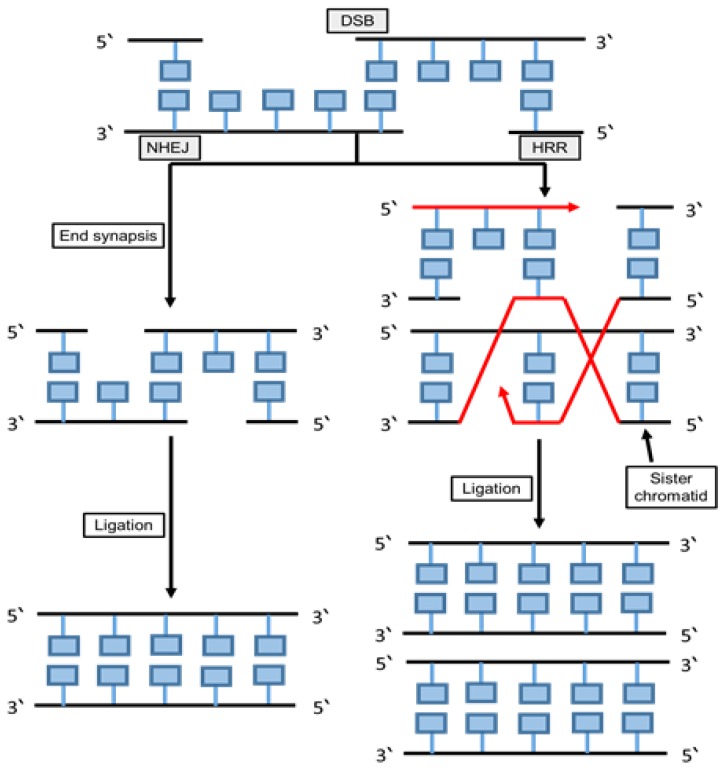
Non-homologous end joining and homologous recombination repair. The schematic diagram summarizes the main components and the mechanisms of the non-homologous end joining and homologous recombination repair pathways. In NHEJ the break ends are directly ligated without the need for a homologous template, as it typically utilizes short homologous DNA sequences called microhomologies to guide repair. In HRR, nucleotide sequences are replaced with two matching molecules of double-stranded or single-stranded nucleic acids, as this pathway requires a homologous sequence to guide repair.

Two mechanisms are mainly used to repair the double strand breaks, including homologous recombination (HR) and non-homologous DNA end joining (NHEJ) as shown in Figure 6. The NHEJ enzymes such as nuclease, DNA polymerases and ligase have a multifunctional and mechanistically flexible effect on DNA repair [111].

#### 6.5.1. Non-Homologous End Joining (NHEJ)

The NHEJ pathway uses a variety of proteins to identify, remove, polymerize and ligate the ends of the DNA. The most difficult damage to repair is the DSBs, which are extremely cytotoxic [112]. Endogenous damage, mainly coming from ROS, causes between 10-50 DSBs per cell every day in tumor cells [113]. The NHEJ pathway is an essential repairing mechanism in DSBs, which is activated in different cell types and cell cycle phases [114]. In mammalian cells, NHEJ is the main pathway to repair DSBs generated from IR, which might cause chromosomal translocations and genomic instability in the case of misrepaired DNA [115]. Anticancer drugs target cytotoxic targets such as type II topoisomerases enzyme which are important for crucial DNA mechanisms (e.g., cut DNA strands). A previous study has shown that topoisomerase IIα and IIβ enzymes have a genotoxic effect and cause particular chromosomal translocations in leukemia. They play an important role in affecting the human cell survival and the efficiency of cancer chemotherapy [116]. The major proteins in NHEJ are KU70 (XRCC6), KU80 (XRCC5), dependent protein kinase catalytic subunit (DNA-PKcs), XRCC4-XLF and ligase 4. However, identifying the DSBs by ATM and MRN complex could be an early stage of NHEJ repair pathway [117]. Enzymes of the family PI3K-related protein kinase (PIKK) in humans include several members such as DNA-PKcs, ATM, ATR and mTOR [118]. Several studies find that DNA-PKcs inhibitors prevent DSBs re-joining and stimulate cytotoxicity [119]. However, any defect in the NHEJ pathway causes a sensitivity to ionizing radiation and excision of lymphocytes [120].

#### 6.5.2. Homologous Recombination Repair (HRR)

HRR is a complicated process pathway that activates to repair DSB damages. This pathway has various proteins, and this repairing mechanism takes place in S and G2 phases in the cell cycle [114]. A limited and critical part of DSBs is the repairing by the HR pathway, because this mechanism is highly accurate and enables the repair of collapsed replication forks, single ended DSBs and interstrand crosslinks (ICLs) [121]. HRR is essential for re-establishing genomic stability. Mutation in one or more genes encoding for HRR proteins can lead to a dysfunction of the whole pathway [122]. However, several tumor-suppressor genes are involved in the HPP pathway, such as BRCA1, BRCA2 and ATM. DSBs are created by IR and topoisomerase I poisons (Camptothecin, Irinotecan and Topotecan), and such damage occurs more in the tumors with a defect in HRR pathway which might improve the efficiency of cytotoxic drugs [123]. The crucial steps in HRR are the activation of RAD51 phosphorylation and the accumulation of RAD51, which is dependent on the proto-oncogene ABL1, and also the ABL1 inhibitor that makes cells more responsive to crosslinking agents (exogenous or endogenous agents affected DNA) and IR [124,125].

The induction of DNA damage and the defect of the DNA damage response (DDR) are responsible for genetic instability, probably associated in the pathogenesis of monoclonal B-cell lymphocytosis (MBL) and chronic lymphocytic leukemia (CLL) [126]. However, excision repair is the repair pathway in CLL as a response to DNA alkylation, and by increasing the level of repair the cells become resistant to alkylating agents [127]. In the future, targeting the DNA repair mechanism could be essential for facilitating the combination of nucleoside analogues and increasing the efficacy of drugs, leading to increased cytotoxicity.

## 7. Cell Cycle as a Checkpoint in DNA Damage

Cell cycle checkpoints are control mechanisms that regulate the order, integrity and fidelity of the cell cycle. These include ensuring correct replication of chromosomes, and their accurate segregation at mitosis [128]. Chromosomal segregation and cell division occur in the G2/M phases of the cell cycle [129]. Protein phosphorylation of signal transducers, mediators and effectors (e.g., p53) induce cell cycle arrest at the G1/S, intra-S or G2/M checkpoints until DNA repair is complete.

The cell cycle has various checkpoints that can be activated in the presence of DNA damage. These checkpoints are responsible for permanent cell cycle arrest or apoptosis of unrepaired DNA damage. However, the repaired cells are progressing to further stages of the cell cycle [130].

## 8. Effects of Chemotherapy or Radiation in Cancer Treatments

Cancer chemotherapy and radiotherapy are designed to cause apoptosis in cancer cells by inducing catastrophic DNA damage such as DSBs. Traditional therapeutic strategies have been developed based on DNA damage response properties of cancer cells that often have specific abnormalities in the pathway [54]. The abnormal expression of a particular DDR protein can be used as a biomarker of therapy resistance, especially when the damage is recognized and misrepaired by intrinsic DNA repair pathways [131].

Chemotherapeutic agents induce DNA damage and cancer cell death via immunogenic cell death, apoptosis and other forms of non-apoptotic death including senescence, mitotic catastrophe and autophagy [132]. Radiotherapy is generally an immune-stimulatory process that causes immunogenic cell death, inflammatory reactions and recruitment of T cells to the tumor microenvironment. Radiotherapy causes lysis of cancer cells. Release of tumor-associated antigens attracts T-cells and dendritic cells and elicits an anti-tumor response [133].

Exposure to chemotherapy can cause several early and late long-term toxicities including ovarian failure (with resultant infertility and sexual dysfunction), bone loss, weight gain, neurotoxicity, neurocognitive changes, cardiac toxicity and secondary malignancy. Such effects have the potential to reduce quality of life and overall health status. Understanding such chemotherapy-related toxicities is of utmost importance [134].

Whilst the effects of cytotoxic chemotherapy on normal bystander cells are widely studied, the specific effects of treatments on cancer genomes are also of importance. Persistence of DNA abnormalities introduced into cancer cells (mutations and chromosomal aberrations) can result in further genomic instability. A similar outcome is also envisaged for radiotherapy. Hence, further studies are needed to understand the long-term effect of radiotherapy and chemotherapy [135,136].

DNA damage and misrepair can persist within normal bystander cells as well as cancer cells, leading to clonal evolution with more aggressive features. Such abnormalities include the formation of abnormal nuclear bodies called micronuclei [137].

## 9. Potential Biomarkers of Chromosomal Abnormalities

Micronuclei (MN) are small extranuclear bodies formed during cell division when the chromosome or a part of the chromosome fails to join the mitotic spindle during M phase [138]. MN are formed spontaneously or induced by chromosomal breaks that form an acentric/whole chromosome fragment. These chromosome fragments are not incorporated into the main nucleus during the restructure of the nuclear envelope around two daughter cells at telophase. Thus, they encapsulate and break up into small nuclear fragments called micronuclei [139,140].

Several studies have described the effects of the exposure to genotoxic agents that result in chromosomal aberrations and genomic instability of cancer cells leading to clonal evolution and progression [141]. Genotoxic agents can induce formation of MN [142]. Identifying MN is an effective method for determining genotoxic effects of chemotherapy and radiotherapy [143].

In cancer cells, mutated p53 alleles lead to reduced apoptosis. It has been shown that the cells with mutated p53 formed more MN after being treated with chemotherapy or irradiation compared to the cancerous cells with wild-type p53 gene. Thus, p53 protein expression is essential for the balance between cell cycle arrest, DNA repair and apoptosis induction [144].

Nuclear anomalies are initiated by structural errors in a chromosome or are due to an abnormal number of chromosomes. Such anomalies include nucleoplasmic bridges (NPB) and nuclear buds (NBUD) and are biomarkers of genotoxic events and manifestations of chromosomal instability that often indicate cancer risk. Genetic damage events such as MN, NPB and NBUD provide valid measures of misrepaired DNA breaks [145]. MN, NPB and NBUD formation could be due to multiple molecular mechanisms. This is supported by a study that reported that increased frequencies of MN, NPB and NBUD in lymphocytes are associated with higher levels of DNA damage [146].

## 10. Clonal Evolution in Cancer

**Figure 7:**
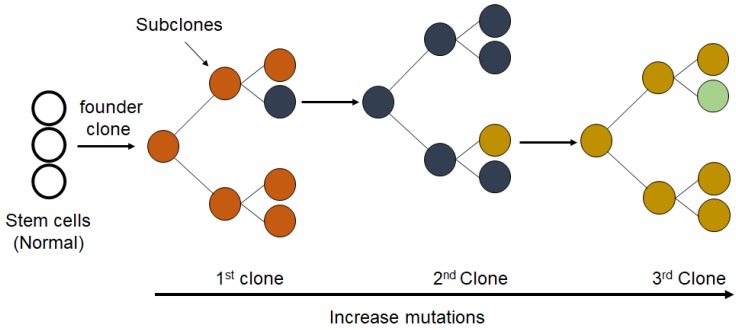
Proposed mechanism for the clonal evolution of cancer. The clonal expansion of a population of mutated cells (cancer cells) from an individual single-cell causes tumor heterogeneity in pathology and molecular profiles with acquired genetic and epigenetic changes.

Clonal evolution can contribute to the development of human cancers and the response to treatment [147]. Chemotherapy can stimulate clonal evolution, metastasis and relapse of tumors in about 20% of cases (Figure 7) [148].

Toxicity associated with chemotherapy is a major therapeutic challenge that is caused by chemotherapy-induced DNA damage and inflammation. Chemotherapy causes selective pressures that create tumor heterogeneity and subsequent clonal evolution [149]. Target therapies are improving the outcomes of many cancers with fewer side effects. Whether such specific treatments can also cause DNA damage, genomic instability and chromosome aberrations is less clear.

The clonal evolution that causes genomic instability could be responsible for more aggressive behavior of cancer cells [150]. This may manifest as higher frequencies of MN due to genomic instability in parallel with the accumulation of mutations. Serial measurements of MN could be useful to monitor levels of DNA damage with various treatments and provide evidence for clonal evolution.

## 11. Conclusions

Investigating DNA damage is essential in the diagnosis and prognosis of several cancers. The formation of some important biomarkers in the cells such as micronuclei (MN), nucleoplasmic bridges (NPB) and nuclear buds (NPB) can be used as an indicator of DNA damage due to exposure to cytotoxic or DNA damaging agents [151]. However, the levels of DNA damage and the efficiency of DNA repair mechanisms affect the frequency of the DNA damage markers.

During the past decades, PARP inhibitors have been combined with single-agent therapies to improve treatment efficacy. This combination is used in the treatment of ovarian cancer and breast cancer with BRCA mutations. Several clinical trials indicated that this particular procedure in treatment is preferable, especially after investigating several predictive biomarker populations [152].

Well-managed mechanisms are responsible for maintaining genome stability and repair of damaged DNA. Genomic instability leads to defects in the normal cellular cells, causing a tendency to convert into cancer cells, which may also occur due to a mutation in the repairing genes [153]. Recently, the development of poly (ADP-ribose) polymerase inhibitor as the first ‘synthetic lethal’ medicine for patients with BRCA-mutant cancers is one of the big challenges to provide patients with a novel targeted therapeutic for cancer cells [154]. The synthetic lethal interactions are utilized by these kinds of therapy to design personalized medicine which depends on the patient’s molecular profiling and behavior of cancer cells. Several benefits of using patient profiling starting from the determination of drug resistance after relapse, could also help in adjusting the therapeutic dose [155]. Precision medicine is a new type of treatment that plays a critical role in selecting the most appropriate therapy at the suitable time, as it only succeeds in targeting the DNA repair pathway in cancer. This treatment strategy is based on a unique genetic background, environment and lifestyle for the individual [156].

The innovative diagnostic methods including DNA damage analysis are improving gradually, especially for precision medicine, and help in analyzing a large amount of new potential biomarkers leading to facilitation of the detection of early disease stages and disease prognosis. These biomarkers are ones such as phosphorylated histone 2Ax (γH2AX), 8-hydroxy-2′-deoxyguanosine (8-OHdG) or 8-oxo-7,8-dihydro-2’-deoxyguanosine (8-oxodG) [157]. The novel makers are still limited in their current use, although all of this is scientific progress [158]. In the context of precision medicine, γH2AX is considered as one of the most promising markers for DNA double strand breaks (DSB) [159]. Finally, the application of precision medicine is the most progressively developed field which depends on the improvement in DNA damage analysis and the investigation of novel markers [159].
